# Absolute quantification of microRNA miR-875-5p in temporal artery biopsies and its biomarker potential for giant cell arteritis

**DOI:** 10.3389/fimmu.2026.1676244

**Published:** 2026-02-10

**Authors:** Luka Bolha, Elvisa Smajlović, Alojzija Hočevar

**Affiliations:** 1 Institute of Pathology, Faculty of Medicine, University of Ljubljana, Ljubljana, Slovenia; 2 Department of Rheumatology, University Medical Centre Ljubljana, Ljubljana, Slovenia; 3 Faculty of Medicine, University of Ljubljana, Ljubljana, Slovenia

**Keywords:** biomarker, digital PCR, giant cell arteritis, microRNA, temporal artery biopsy

## Abstract

**Objectives:**

To perform absolute quantification of miR-875-5p expression in temporal artery biopsies (TABs) of patients with giant cell arteritis (GCA), associate miR-875-5p copy number with histological and clinical characteristics of patients with GCA, and evaluate the diagnostic value of absolute copy number of miR-875-5p in GCA.

**Methods:**

The study included formalin-fixed, paraffin-embedded TABs of 45 treatment-naïve clinically proven patients with GCA, and 19 non-GCA controls. Of the included patients with GCA, 29 had histologically positive and 16 histologically negative TABs. Expression of miR-875-5p was assessed through utilization of quantitative real-time PCR (qPCR) and absolute quantification by digital PCR (dPCR).

**Results:**

We determined significantly higher absolute copy number of miR-875-5p in histologically positive TABs of patients with GCA, compared to histologically negative TABs of GCA and non-GCA patients, which significantly correlated with the majority of TAB histopathological parameters and several clinical characteristics of patients with GCA. Notably, we showed a good diagnostic performance of absolute copy number of miR-875-5p in discriminating patients and controls, depending on the extent of vessel wall inflammation and remodeling in affected temporal arteries.

**Conclusion:**

Our study revealed that absolute quantification of miR-875-5p expression holds the potential to serve as a supporting biomarker in assessing vessel wall inflammation and remodeling in patients with GCA. Moreover, our results indicate the applicability of absolute quantification by dPCR in detecting low-abundance miRNAs in GCA-affected arterial tissue, whose limiting amounts hinder the utilization of classical qPCR.

## 1 Introduction

Giant cell arteritis (GCA) is a primary systemic vasculitis affecting large- and medium-sized arteries, characterized by a transmural inflammation and subsequent remodeling of the vascular wall, and related ischemic manifestations, occurring in individuals aged 50 years and older (1, 2). Current understanding of GCA pathogenesis and pathophysiology, suggests that microRNA (miRNA) dysregulation plays a crucial contributing factor to the shaping of the inflammatory milieu and inflammatory-driven vessel wall remodeling process in affected arteries (3). In line, a set of aberrantly expressed miRNAs has been associated with several histopathological and clinical characteristics of GCA, implying to their biomarker potential in capturing vessel wall inflammation (3), whose persistent and self-renewing nature currently hinders the management of GCA (4). Based on the results of our previously performed quantitative real-time PCR (qPCR)-based miRNA expression profiling experiments in temporal artery biopsies (TABs) from treatment-naïve patients with GCA (5), a putative biomarker potential of eight miRNAs emerged (including miR-146a-3p, miR-146b-3p, miR-147a, miR-147b-3p, miR-211-5p, miR-548a-5p, miR-642a-5p and miR-875-5p), as the detection of their expression appeared specific to GCA, and was limited only to histologically positive TABs. Notably, data on these eight miRNAs was not presented in the published study (5). Following preliminary qPCR testing, seven miRNAs were excluded from further analysis, due to stochasticity in their expression, quantification cycle (Cq) values ≥ 35, and non-specific amplification of the cDNA template and/or primer dimers as determined by qPCR melt curve analysis. As such, miR-875-5p was the only miRNA that passed the above-mentioned exclusion cut-off criteria and was deemed suitable for a further assessment of its putative biomarker potential to discriminate TABs on a molecular level.

Therefore, the aim of this study was to validate the expression of miR-875-5p in TABs of treatment-naïve clinically proven patients with GCA and TABs from control non-GCA patients, through utilization of qPCR and absolute quantification by digital PCR (dPCR), and thus to verify its putative GCA-related specificity. In addition, we evaluated the diagnostic value of absolute copy number of miR-875-5p in TABs of patients with GCA, and assessed the association between the expression of miR-875-5p, and several histopathological and clinical characteristics of patients with GCA. Moreover, we put the aberrant miR-875-5p expression into the pathophysiological context of GCA.

## 2 Materials and methods

### 2.1 Patients

This retrospective study included TABs of 45 clinically proven newly diagnosed patients with active GCA, comprising 29/45 (64%) histologically positive TABs and 16/45 (36%) histologically negative TABs. The control non-GCA group included TABs of 19 age-matched patients with a clinical suspicion of GCA, which was refuted after a thorough clinical and histopathological evaluation, and a six-month follow-up. Notably, all 45 patients diagnosed with GCA and 19 non-GCA controls included in our current study were also included in two of our previous studies (5, 6). These 45 patients and 19 controls are overlapping between all three studies, whereas we were compelled to exclude several cases from studies Bolha et al., 2020 and 2021 (5, 6) due to limiting amounts of isolated RNA and/or limiting amount of stored TAB material. Characteristics of patients included in the study are presented in **Table 1**.

**Table 1:** Patient characteristics.

Characteristic	GCA	GCA	Non-GCA	*p*-value^a^
	TAB-positive	TAB-negative	TAB-negative	
Number of patients	29	16	19	
Sex [male/female]; n (% female)	7/22 (76)	8/8 (50)	4/15 (79)	0.082
Age at biopsy [years]; median (range)	73 (66–89)	73 (57–92)	74 (58–87)	0.255
Constitutional symptoms; n (%)	21/29 (72)	11/16 (69)	N/A	0.797
PMR; n (%)	5/29 (17)	3/16 (19)	N/A	0.900
New headache; n (%)	27/29 (93)	11/16 (69)	N/A	**0.033**
Jaw claudication; n (%)	17/29 (59)	0/16 (0)	N/A	**< 0.001**
GCA relapse; n (%)	11/29 (38)	4/16 (25)	N/A	0.384
Visual disturbances; n (%)	9/29 (31)	4/16 (25)	N/A	0.672
Permanent visual loss; n (%)	2/29 (7)	0/16 (0)	N/A	0.288
Clinically altered TA; n (%)	26/29 (90)	4/16 (25)	N/A	**< 0.001**
Halo; n (%)^b^	27/28 (96)	9/16 (56)	N/A	**0.001**
Stenosis/occlusion; n (%)^b^	22/28 (79)	5/16 (31)	N/A	**0.002**

GCA, giant cell arteritis; TAB, temporal artery biopsy; PMR, polymyalgia rheumatica; TA, temporal artery; N/A, not applicable.

a Data between TAB-positive and TAB-negative patients with GCA were evaluated using the Mann-Whitney *U* test. A *p*-value of < 0.05 was considered statistically significant (marked in bold).

b Determined by color Doppler ultrasonography examination of TAs. Data was available for 28 TAB-positive patients with GCA.

All TABs were obtained for diagnostic purposes, and were histopathologically evaluated, routinely fixed in 10% neutral buffered formalin, embedded in paraffin and archived at the Institute of Pathology (Faculty of Medicine, University of Ljubljana). TABs were collected between September 2011 and December 2015. The diagnosis of GCA was based on clinical, laboratory, imaging (the presence of the halo sign on a color Doppler ultrasonography scan of temporal arteries or a positive positron emission tomography with computed tomography (PET/CT) scan) and/or histological (a positive TAB) findings, and a six-month follow-up. All GCA patients fulfilled the American College of Rheumatology (ACR) 1990 criteria for the classification of GCA (7), whereas 41/45 (91%) GCA cases also fulfilled the 2022 ACR/European Alliance of Associations for Rheumatology (EULAR) classification criteria for GCA (8). All patients had clinical suspicion of GCA and were subjected to the fast-track GCA pathway, where a TAB or vascular ultrasound was performed within 24 h from referral to the rheumatologist. Therapy commenced when the diagnosis of GCA was proven, on the same day of a diagnostic procedure. All included patients were treatment-naïve prior to the TAB. The study was approved by the National Medical Ethics Committee of the Republic of Slovenia [approval Nos. 65/01/17, 0120-215/2020 and 0120-118/2023/6] and was performed in accordance with the Declaration of Helsinki.

### 2.2 RNA isolation and reverse transcription

Isolation of total RNA was performed manually from 10 10-µm thick sections of formalin-fixed, paraffin-embedded (FFPE) TAB samples, with an AllPrep^®^ DNA/RNA FFPE Kit (80234, Qiagen, Germany), as described previously (5).

Reverse transcription and cDNA synthesis was performed with a miRCURY LNA RT Kit (339340, Qiagen, Germany) in 10 µl reaction volumes, according to the manufacturer’s protocol. Detailed information on the procedure is available elsewhere (5).

### 2.3 Quantitative real-time PCR analysis

qPCR experiments were performed to assess the expression of miR-875-5p in TABs, and to evaluate the suitability of an individual TAB sample for subsequent dPCR analysis. Analysis was performed in 10 µl reaction mixtures in duplicate, with a miRCURY SYBR Green PCR Kit (339347, Qiagen, Germany) and miRCURY LNA miRNA PCR Assays (339306; Qiagen, Germany) on a QuantStudio™ 7 Pro Real-Time PCR System (Thermo Fisher Scientific, USA), according to the manufacturer’s protocol, and as described previously (9). The UniSp6 primer assay was used as a reverse transcription positive control. As miR-875-5p expression levels appeared to be limiting at 1:60 cDNA dilution, a dilution that was used in qPCR-based miRNA expression profiling experiments (5), we performed qPCR analyses using 3 µl of 1:30 (0.1 ng) dilution of the cDNA template for all included samples.

**Figure 1:**
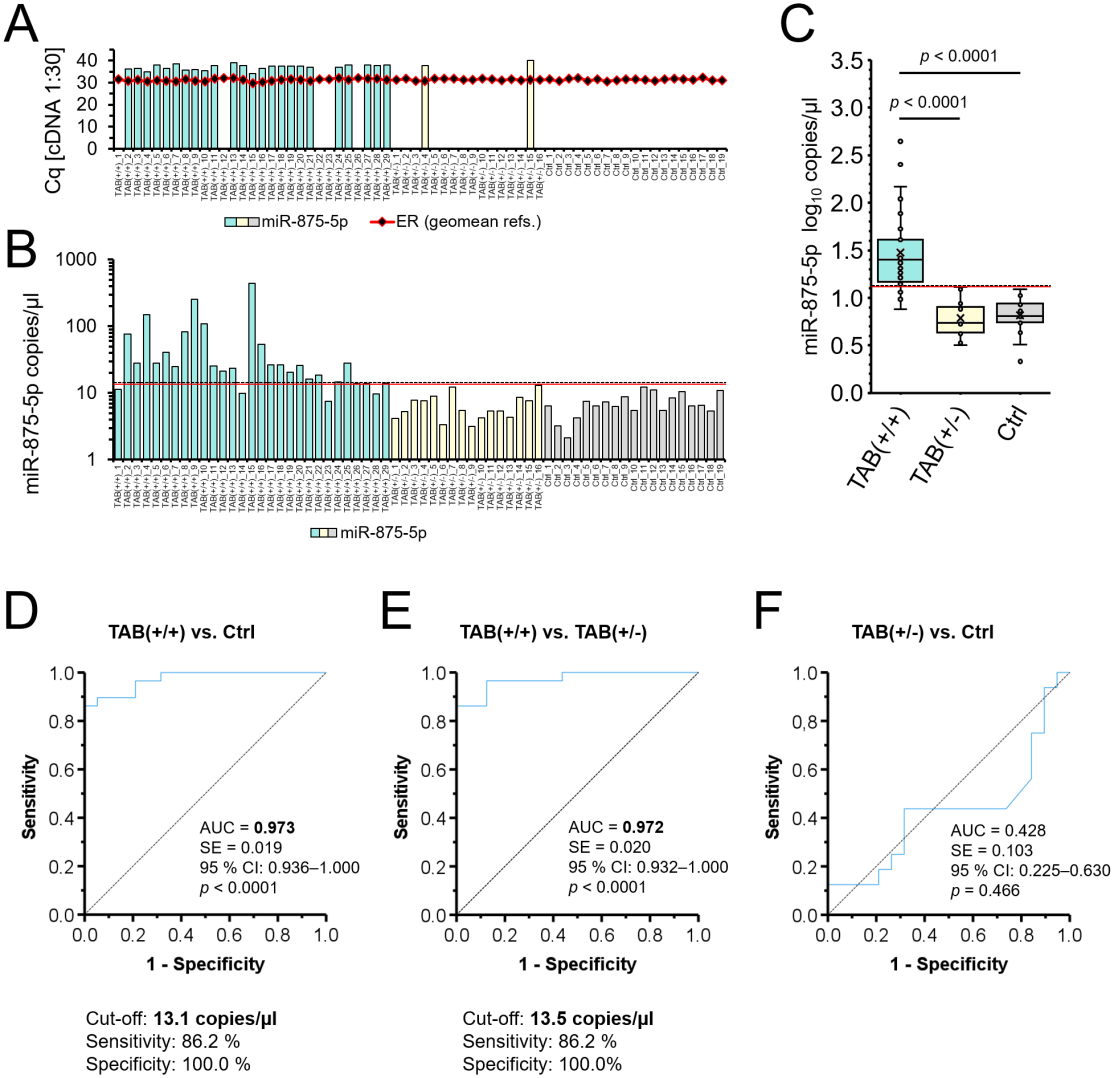
Expression of miR-875-5p and its diagnostic value. **(A–C)** Expression of miR-875-5p in histologically positive TABs of patients with GCA [TAB(+/+); n = 29], histologically negative TABs of patients with GCA [TAB(+/-); n = 16], and histologically negative TABs of non-GCA patient controls [Ctrl; n = 19]. Samples from the TAB(+/+), TAB(+/-) and Ctrl groups are indicated in light turquoise, light yellow and grey, respectively. **(A)** Expression of miR-875-5p at 1:30 cDNA dilution as determined by quantitative real-time PCR (qPCR). The bars represent the determined quantification cycle (Cq) values for miR-875-5p, and black dots within the red line geometric means of Cq values of reference miRNAs miR-362-3p and miR-500a-5p. **(B)** Absolute copy number of miR-875-5p as determined by digital PCR (dPCR). Each bar represents an absolute copy number of miR-875-5p per µl of undiluted cDNA, determined for an individual sample. **(C)** Summarization and statistical evaluation of differences in the absolute copy number of miR-875-5p between patient groups. Each dot represents the log_10_ miR-875-5p copy number per µl of an individual sample. The horizontal line within the boxplot denotes the median, and the horizontal border lines the interquartile range. The symbol × denotes the average. Data were evaluated using the Mann-Whitney *U* test. An adjusted *p*-value of < 0.05 was considered statistically significant. **(B, C)** The red and black dotted lines represent optimal cut-offs to distinguish TAB(+/+) group from the Ctrl group [13.1 copies/µl; **(D)**], and TAB(+/+) group from the TAB(+/-) group [13.5 copies/µl; **(E)**], respectively. **(D–F)** Receiver operating characteristic (ROC) curves for absolute copy number of miR-875-5p per µl: differentiation between TAB(+/+) and Ctrl groups **(D)**, differentiation between TAB(+/+) and TAB(+/-) groups **(E)**, and differentiation between TAB(+/-) and Ctrl groups **(F)**. The area under the ROC curve (AUC), standard error (SE), 95% confidence interval (CI) and a *p*-value are presented for each ROC curve. An optimal cut-off value, sensitivity and specificity were determined by calculating the Youden index. A *p*-value of < 0.05 was considered statistically significant.

All samples were deemed suitable for dPCR, based on the expression of miR-362-3p and miR-500a-5p, previously utilized as endogenous controls for data normalization in qPCR experiments on GCA and non-GCA TABs (5, 6), which was comparable between all assessed samples, including geometric means of their Cq values (**Supplementary Figure S1**; **Figure 1A**). Primer assays included in qPCR and/or dPCR analyses are listed in **Supplementary Table S1**.

### 2.4 Digital PCR analysis

All dPCR analyses were conducted in accordance with the Minimum Information for Publication of Quantitative Digital PCR Experiments for 2020 (The Digital MIQE Guidelines Update) (10) guidelines. Absolute quantification of miR-875-5p expression in TABs was performed on a QIAcuity One Digital PCR System (Qiagen, Germany) with EvaGreen PCR Master Mix (250112, Qiagen, Germany), using miRCURY LNA miRNA PCR Assays (339306; Qiagen, Germany), according to the manufacturer’s protocol. Analyses were performed in 40 µl reaction mixtures on QIAcuity Nanoplates 26k (24-well) (250001, Qiagen, Germany). Each singleplex dPCR reaction mixture contained 13.3 µl of 3× EvaGreen PCR Master Mix, 4 µl of 10× miRCURY LNA PCR Assay, 12.7 µl of RNase-free water and 10 µl of cDNA template diluted 1:5 (2.0 ng). A no-template control (NTC) was included into each dPCR run. Based on the preliminary dPCR testing, where 1:30 cDNA dilutions yielded an insufficient amount of positive partitions per each well of the QIAcuity Nanoplate, cDNA template diluted 1:5 was used for final dPCR analyses.

PCR cycling conditions were as follows: i) initial heat activation at 95°C for 2 min; ii) 55 two-step cycles of denaturation at 95°C for 15 s and combined annealing/extension for 1 min at 60°C; and iii) cooling down for 5 min at 40°C. Raw data was analyzed using a QIAcuity Software Suite v3.1.0.0 (Qiagen, Germany) with activated green channel, according to the manufacturer’s user manual. The exposure time and gain used for Nanoplate partition imaging was set at 240 ms and 3, respectively. The fluorescence threshold was set manually and unified across all analyzed samples and controls (at fluorescence intensity (RFU) of 88), to ensure comparable fluorescence intensity cut-offs (**Supplementary Figure S2**). The final concentration of miR-875-5p in TABs was expressed as miRNA copies per µl of undiluted cDNA (1 ng/µl).

### 2.5 Data on TAB histopathology

For the purpose of our current study, to assess the association between absolute copy number of miR-875-5p and TAB histopathology, we included data on quantitatively assessed TAB histopathological parameters obtained from our previously performed studies (5, 6). These parameters comprised the intima-media thickness, the ratio between the intima and media thickness, and densities of CD3^+^, CD4^+^, CD8^+^ T cells, CD20^+^ B cells, CD68^+^ macrophages, multinucleated giant cells (MGCs) and eosinophil granulocytes per mm^2^, for all 45 included patients with clinically proven GCA. For histopathological evaluation, 4-µm thick sections were cut from FFPE tissue blocks and stained with hematoxylin and eosin (HE). The extent of intimal hyperplasia was evaluated quantitatively with an eyepiece micrometer, where the thickness of the intima and the media were measured separately. Densities of lymphocyte subtypes and macrophages were determined immunohistochemically and by using an image analysis system (Cell and Tissue Analysis, Leica, Germany). Density of CD3^+^, CD4^+^, CD8^+^ T cells and CD20^+^ B cells was expressed as an average number of cells per mm^2^, whereas a 4-tier scoring system was adopted for CD68^+^ macrophages due to inability to count these cells in heavy infiltrates on account of indistinct borders. The number of MGCs and eosinophil granulocytes per mm^2^ was evaluated histopathologically on HE-stained FFPE sections. A detailed description of the performed procedures is presented elsewhere (5, 6).

### 2.6 Statistical analysis

Statistical analyses were performed with IBM SPSS Statistics 27.0 software (IBM Corporation, USA). The Q–Q plots, Shapiro-Wilk and Kolmogorov-Smirnov tests were used to assess the normality of data distribution. Following a non-normal data distribution, the Mann-Whitney *U* test was used to assess differences in absolute miRNA copy number between patient groups, and Spearman’s (*ρ*) correlation coefficients to evaluate associations between parameters. The Bonferroni correction method was used to adjust the obtained *p*-values due to multiple performed comparisons (n = 3). The receiver operating characteristic (ROC) curves were used to evaluate the performance of miR-875-5p copy number in discriminating between TABs of patients with GCA and controls. The area under the ROC curve (AUC), standard error (SE), 95% confidence interval (CI) and a *p*-value were determined for each ROC curve. An optimal cut-off value (copies of miR-875-5p per µl), sensitivity and specificity were determined by calculating the Youden index (Youden’s J statistics). All tests were two-tailed and a *p*-value of < 0.05 was considered statistically significant in all cases.

## 3 Results

### 3.1 Expression of miR-875-5p in TABs of patients with GCA and controls

Results of our qPCR analyses revealed that miR-875-5p was expressed in 24/29 (83%) histologically positive TABs of patients with GCA (all Cq > 34), whereas miR-875-5p was amplified only in 2/16 (13%) histologically negative TABs of patients with GCA (both Cq > 37) and in no histologically negative TABs of non-GCA patient controls (**Figure 1A**). Notably, geometric means of Cq values of reference miRNAs (miR-362-3p and miR-500a-5p) were within the range of 2.5 Cqs between all assessed TAB samples at 1:30 cDNA dilution, indicating to comparable efficiency of cDNA synthesis between samples and accurate preparation of cDNA dilutions (**Figure 1A**).

Due to restricted expression of miR-875-5p in histologically negative TABs of GCA and non-GCA patients and inadequate sensitivity of qPCR *per se* to detect miR-875-5p in the majority of these samples, we further assessed the expression of miR-875-5p by utilizing dPCR. Overall, a median of 25.3 copies/µl was determined in histologically positive TABs of patients with GCA (range 7.6–442.4 copies/µl), a median of 5.4 copies/µl in histologically negative TABs of patients with GCA (range 3.2–13.0 copies/µl) and a median of 6.5 copies/µl in histologically negative TABs of non-GCA controls (range 2.1–12.3 copies/µl) (**Figures 1B, C**). Absolute copy number of miR-875-5p significantly distinguished TAB-positive patients with GCA from TAB-negative GCA patients and controls (both *p* < 0.0001) (**Figure 1C**).

### 3.2 Biomarker potential of absolute copy number of miR-875-5p

The diagnostic value of absolute copy number of miR-875-5p to distinguish between TABs of patients with GCA and controls was assessed by performing the ROC curve analysis (**Figures 1D–F**). As revealed, miR-875-5p yielded a high significant AUC of 0.973 (95% CI: 0.94–1.00, *p* < 0.0001) and an optimal cut-off at 13.1 copies/µl with 86% sensitivity and 100% specificity in discriminating TAB-positive patients with GCA from TAB-negative non-GCA controls (**Figure 1D**; optimal cut-off depicted as a red line in **Figures 1B, C**). Similarly, miR-875-5p yielded a comparable significant AUC of 0.972 (95% CI: 0.93–1.00, *p* < 0.0001) and an optimal cut-off at 13.5 copies/µl with 86% sensitivity and 100% specificity in discriminating TAB-positive from TAB-negative patients with GCA (**Figure 1E**; optimal cut-off depicted as a black dotted line in **Figures 1B, C**). In contrast, poor diagnostic performance was determined for miR-875-5p copy number in distinguishing TAB-negative patients with GCA from non-GCA controls, yielding an AUC of 0.428 (95% CI: 0.23–0.63, *p* = 0.466) (**Figure 1F**).

### 3.3 Expression of miR-875-5p in relation to histological and clinical characteristics of patients with GCA

**Figure 2:**
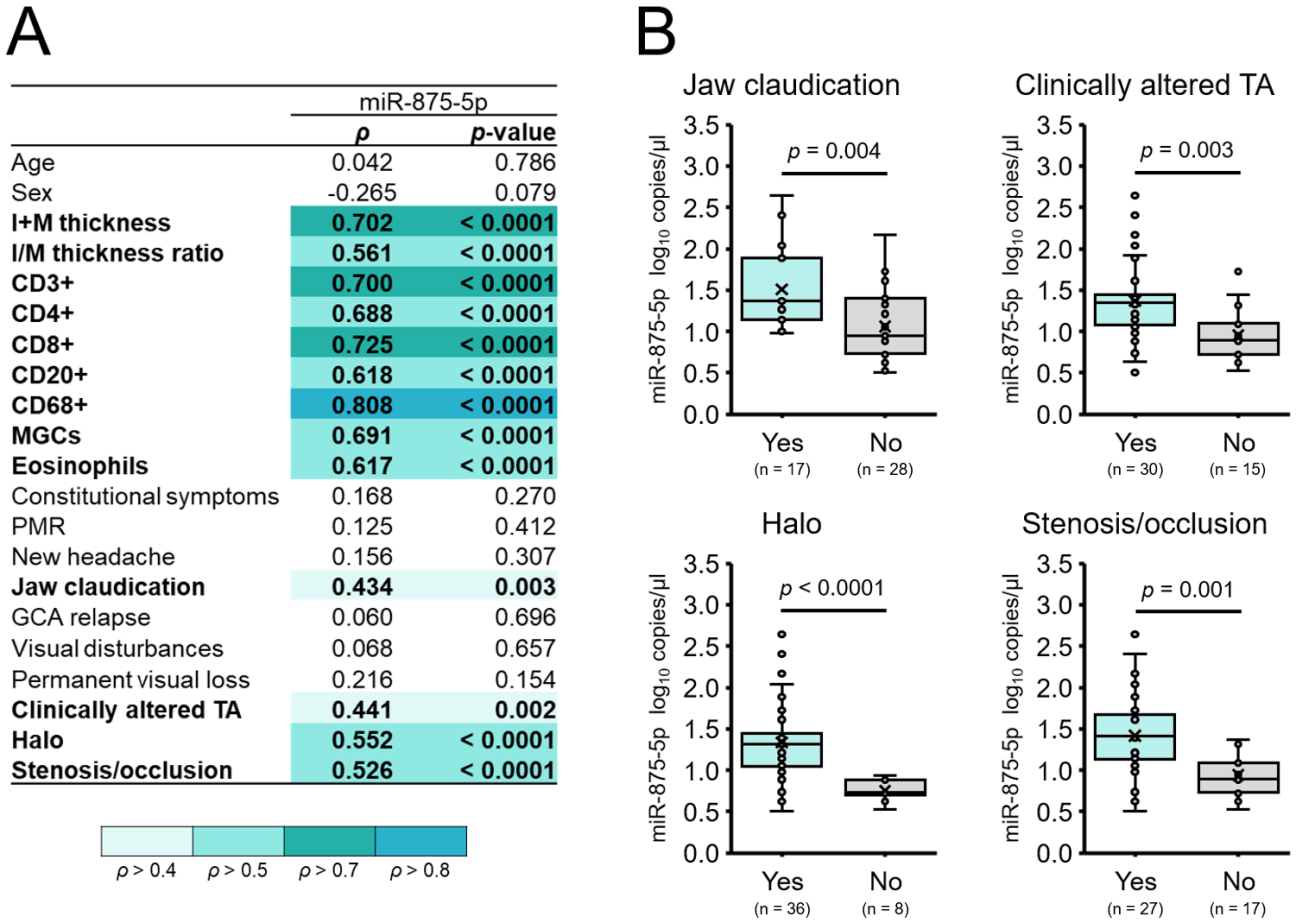
Expression of miR-875-5p in relation to characteristics of patients with GCA. **(A)** Spearman’s correlation coefficients (*ρ*) between the absolute copy number of miR-875-5p per µl, and characteristics of patients with GCA (n = 45). A *p*-value of < 0.05 was considered statistically significant (marked in bold). **(B)** Expression of miR-875-5p in patients with, and patients without jaw claudication, clinically altered temporal arteries, the halo sign and stenosis/occlusion. Each dot represents the log_10_ miR-875-5p copy number per µl of an individual sample. The horizontal line within the boxplot denotes the median, and the horizontal border lines the interquartile range. The symbol × denotes the average. Data were evaluated using the Mann-Whitney *U* test. A *p*-value of < 0.05 was considered statistically significant.

Results of Spearman’s (*ρ*) correlation analysis revealed a significant positive correlation between the number of miR-875-5p copies/µl and all nine included quantitatively assessed TAB histopathological parameters of 45 patients with GCA (all *p* < 0.0001). Of these, the intima-media thickness, and densities of CD3^+^, CD8^+^ and CD68^+^ immune cells per mm^2^ showed the strongest positive correlation (all *ρ* ≥ 0.700) (**Figure 2A**).

Notably, absolute copy number of miR-875-5p also significantly positively correlated with the presence of jaw claudication, clinically altered temporal arteries, the halo sign and stenosis/occlusion in patients with GCA (all *ρ* ≥ 0.434; *p* ≤ 0.003) (**Figure 2A**). By assessing miR-875-5p copy number in regard to these four clinical features, we revealed a significantly higher number of miR-875-5p copies/µl in patients with, compared to patients without jaw claudication, clinically altered temporal arteries, the halo sign and stenosis/occlusion (all *p* ≤ 0.004) (**Figure 2B**).

In addition, in a subgroup of 29 TAB-positive patients with GCA, the number of miR-875-5p copies/µl significantly positively correlated with the intima-media thickness (*ρ* = 0.569; *p* = 0.022), density of CD68^+^ macrophages per mm^2^ (*ρ* = 0.418; *p* = 0.047) and the presence of stenosis/occlusion (*ρ* = 0.409; *p* = 0.010) (**Supplementary Table S2**).

## 4 Discussion

In our study, we performed absolute quantification of miR-875-5p expression in TABs of patients with GCA and non-GCA controls, and thus showed that miRNAs present in limiting amounts in GCA-affected arteries, can be effectively quantified by using dPCR. Notably, we revealed that miR-875-5p copy number is significantly higher in histologically positive TABs of patients with GCA, compared to histologically negative TABs of GCA and non-GCA patients. Moreover, we showed that miR-875-5p may serve as a supporting biomarker in capturing the extent of vessel wall inflammation, mainly due to its high specificity, reaching 100% (no false positives) at determined cut-offs, however, with a prerequisite to be combined with other markers to increase sensitivity. Despite its relatively good biomarker potential in addressing the vascular disease component of GCA (i.e., vascular wall inflammation) (4), poor performance of miR-875-5p copy number has been determined in distinguishing TAB-negative patients from TAB-negative non-GCA controls, whereas its prognostic potential for GCA also remains currently unexplored and needs further assessment.

Involvement and function of miR-875-5p in the pathophysiology of vasculitides, including GCA, is currently unknown. Studies have shown that miR-875-5p is a cancer-related miRNA, whose overexpression has been associated with tumor-suppressive function in cervical cancer, hepatocellular carcinoma and gastric cancer, through inhibition of tumor growth, progression and metastasis (11–13). In the context of GCA pathogenesis, miR-875-5p might be implicated in the neurogenic locus notch homolog protein (NOTCH) signaling pathway, since it has been shown to negatively regulate the expression of NOTCH3 in gastric cancer and osteoarthritis (14, 15). Overexpression of miR-875-5p and miR-875-5p-mediated downregulation of NOTCH3 in GCA-affected temporal arteries could thus contribute to the pathogenic vascular smooth muscle cell (VSMC) phenotypic transition into the synthetic state, resulting in the development of intimal hyperplasia and related ischemic manifestations in patients with GCA, as it has been proposed for miR-150 (3). In addition, pro-inflammatory activity of miR-875-5p determined in a gestational diabetes mellitus rat model (16), and negative regulation of anti-inflammatory M2 macrophage polarization by miR-875-3p in gastric cancer (17), suggest that overexpression of miR-875 might at least partly promote the inflammatory-driven vessel wall remodeling process in GCA. However, although the elevated number of miR-875-5p copies in TABs of patients with GCA associated well with the degree of vascular wall remodeling and the inflammatory infiltrate composition, it is unlikely that miR-875-5p belongs to key aberrantly expressed, “disease-driving” miRNAs in GCA (3), mainly due to its limiting copy number even in histologically positive TABs. Nonetheless, according to the above-mentioned studies, we may speculate that miR-875 exerts it function through interaction with other non-coding RNAs (ncRNAs), including miRNAs, circular RNAs (circRNAs) and long non-coding RNAs (lncRNAs) (14, 15, 17), although the exact role and function of miR-875 dysregulation in the pathogenesis of GCA still needs to be determined.

Overall, TABs obtained for diagnostic purposes represent a useful source for researchers to characterize GCA-affected arteries on a molecular level, including the detection of aberrantly expressed miRNAs and the identification of putative miRNA-based biomarkers. However, molecular assessment of TABs is currently still relatively costly and time consuming, and not yet proven for diagnostic purposes in GCA. While spatial miRNA expression patterns could be detected *in situ* by *in situ* hybridization (ISH) assays (e.g., miRNAscope) in a relatively timely manner and could be added to standardized staining procedures, application of such assays would be impractical in the context of an expeditious histopathological evaluation of TABs obtained as a part of an intra-operative frozen section analysis, commonly used in diagnosing GCA. Therefore, although identified dysregulated GCA-related arterial tissue miRNAs exhibit good diagnostic potential in molecular discrimination between different subgroups of patients with GCA and non-GCA controls, they may rather serve as prognostic biomarkers, through their assessment in the circulation of patients with GCA, of course with a prerequisite to demonstrate overlapping altered “tissue-circulatory” expression profiles. As such, given its prominent expression in histologically positive TABs, miR-875-5p might be released into the bloodstream from affected arteries and detected by dPCR in GCA patient’s blood plasma and/or serum, enabling a relatively rapid recognition of GCA relapse. Nevertheless, further studies are needed to confirm such applicability of miR-875-5p detection and its prognostic potential in clinical settings.

In summary, our study shows for the first time that absolute quantification by dPCR may be applicable in detecting low-abundance miRNAs in GCA-affected arterial tissue, whose limiting amounts hinder utilization of classical qPCR, and that absolute copy number of miR-875-5p holds the potential to serve as a supporting biomarker in assessing vessel wall inflammation in patients with GCA. While there is a necessity for additional evaluation of the biomarker validity of miR-875-5p in GCA through inclusion of larger patient cohorts, preferably from different geographic locations, integration of miR-875-5p with other dysregulated GCA-related miRNAs into dPCR-based multiplex miRNA panels may represent a promising strategy to improve the diagnostic and prognostic assessment of GCA. Furthermore, utilization of specific multi-miRNA biomarkers on bodily fluids, such as blood serum and/or plasma, might eventually anticipate GCA relapses, considerably improving the management of GCA.

## Data Availability

The original contributions presented in the study are included in the article/**Supplementary Material**. Further inquiries can be directed to the corresponding author/s.
