# Supplementary material for: Absolute quantification of microRNA miR-875-5p in temporal artery biopsies and its biomarker potential for giant cell arteritis
Source: Front Immunol. 2026 Feb 10;17:1676244. doi: 10.3389/fimmu.2026.1676244 (PMC12929443; doi:10.3389/fimmu.2026.1676244)
Supplement: Supplementary file 1 [file Table1.docx]

***Supplementary Material***

**Supplementary Table S1.** miRNA primer assays.

| miRNA | Catalogue number^a^ | Application |
| --- | --- | --- |
| miR-875-5p | YP00205978 | qPCR, dPCR |
| miR-362-3p | YP00205612 | qPCR |
| miR-500a-5p | YP00204794 | qPCR |
| UniSp6^b^ | YP00203954 | qPCR |

qPCR, quantitative real-time PCR; dPCR, digital PCR.

^a^miRCURY LNA miRNA PCR Assay catalogue number (product number 339306, Qiagen, Germany).

^b^miRCURY LNA miRNA PCR Assay specific for the amplification of the UniSp6 RNA spike-in control, used as a reverse transcription positive control.

**Supplementary Table S2.** Association between the expression of miR-875-5p and characteristics of TAB-positive patients with GCA.

|  | miR-875-5p | |
| --- | --- | --- |
|  | ***ρ*** | ***p*-value** |
| Age | –0.439 | 0.210 |
| Sex | –0.347 | 0.157 |
| **I+M thickness** | **0.569** | **0.022** |
| I/M thickness ratio | 0.463 | 0.054 |
| CD3^+^ T cells | –0.179 | 0.674 |
| CD4^+^ T cells | –0.300 | 0.810 |
| CD8^+^ T cells | 0.159 | 0.284 |
| CD20^+^ B cells | –0.377 | 0.473 |
| **CD68^+^ macrophages** | **0.418** | **0.047** |
| MGCs | 0.415 | 0.312 |
| Eosinophils | 0.309 | 0.342 |
| Constitutional symptoms | 0.116 | 0.201 |
| PMR | –0.035 | 0.652 |
| New headache | –0.146 | 0.449 |
| Jaw claudication | 0.098 | 0.698 |
| GCA relapse | 0.063 | 0.743 |
| Visual disturbances | –0.065 | 0.783 |
| Permanent visual loss | 0.062 | 0.399 |
| Clinically altered TA | –0.045 | 0.552 |
| Halo | 0.322 | 0.095 |
| **Stenosis/occlusion** | **0.409** | **0.010** |

TAB, temporal artery biopsy; GCA, giant cell arteritis; I, intima; M, media; CD, cluster of differentiation; MGC, multinucleated giant cell; PMR, polymyalgia rheumatica; TA, temporal artery.

Spearman’s correlation coefficients (*ρ*) between the absolute copy number of miR-875-5p per µl, and characteristics of patients with GCA with histologically positive TABs (n = 29). A *p*-value of < 0.05 was considered statistically significant (marked in bold).


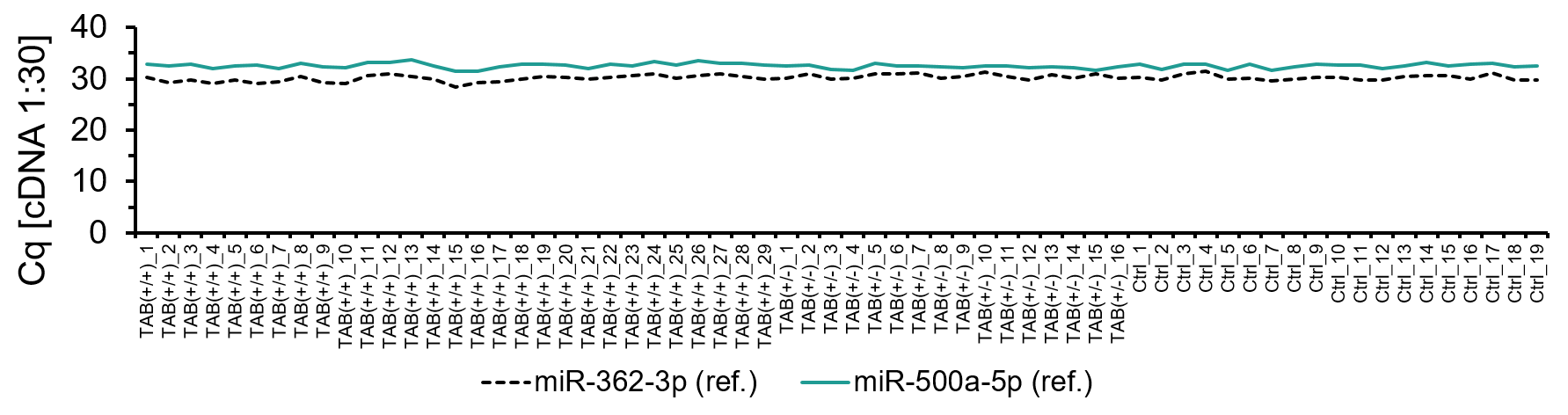


**Supplementary Figure S1.** Expression of miR-362-3p and miR-500a-5p. Expression of reference miRNAs, miR-362-3p and miR-500a-5p, in all 64 TABs included in the study, as determined by quantitative real-time PCR (qPCR). The lines represent the determined quantification cycle (Cq) values at 1:30 cDNA dilution.


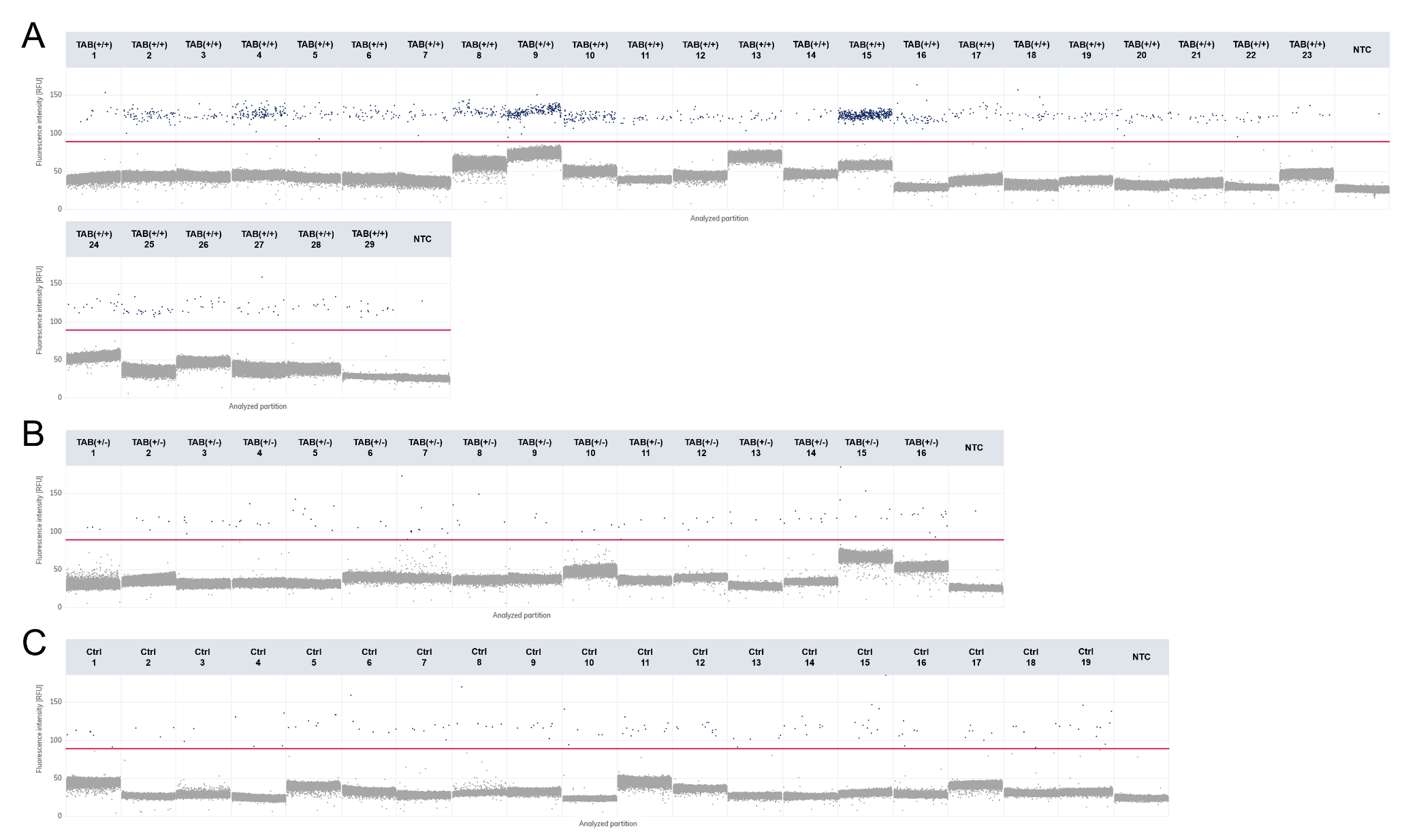


**Supplementary Figure S2.** Data output from the QIAcuity One Digital PCR System. One-dimensional scatter plots, depicting amplification of miR-875-5p in each partition of the QIAcuity Nanoplate, are presented for 29 histologically positive TABs of patients with GCA [TAB(+/+)] **(A)**, 16 histologically negative TABs of patients with GCA [TAB(+/-)] **(B)**, and 19 histologically negative TABs of non-GCA patient controls [Ctrl] **(C)**. The x-axis shows the number of analyzed Nanoplate partitions for each sample (n_total_ = 26,000), and the y-axis fluorescence intensity (relative fluorescence units; RFU) detected in each partition after PCR amplification. Negative partitions (no amplification of miR-875-5p) are presented in grey and positive partitions (amplification of miR-875-5p) in dark blue. Red lines indicate a common fluorescence threshold at 88 RFU. The same no-template control (NTC) is presented for TAB(+/+) samples 24–29 and all 16 TAB(+/-) samples, since these samples were analyzed simultaneously on the same Nanoplate.
